# Supplementary material for: A Core Module of Nuclear Genes Regulated by Biogenic Retrograde Signals from Plastids
Source: Plants (Basel). 2021 Feb 4;10(2):296. doi: 10.3390/plants10020296 (PMC7913978; doi:10.3390/plants10020296)
Supplement: Supplementary file 1 [file plants-10-00296-s001.zip › Grübler et al 2021 Supplementary Figures Revision.pptx]

## Slide 1
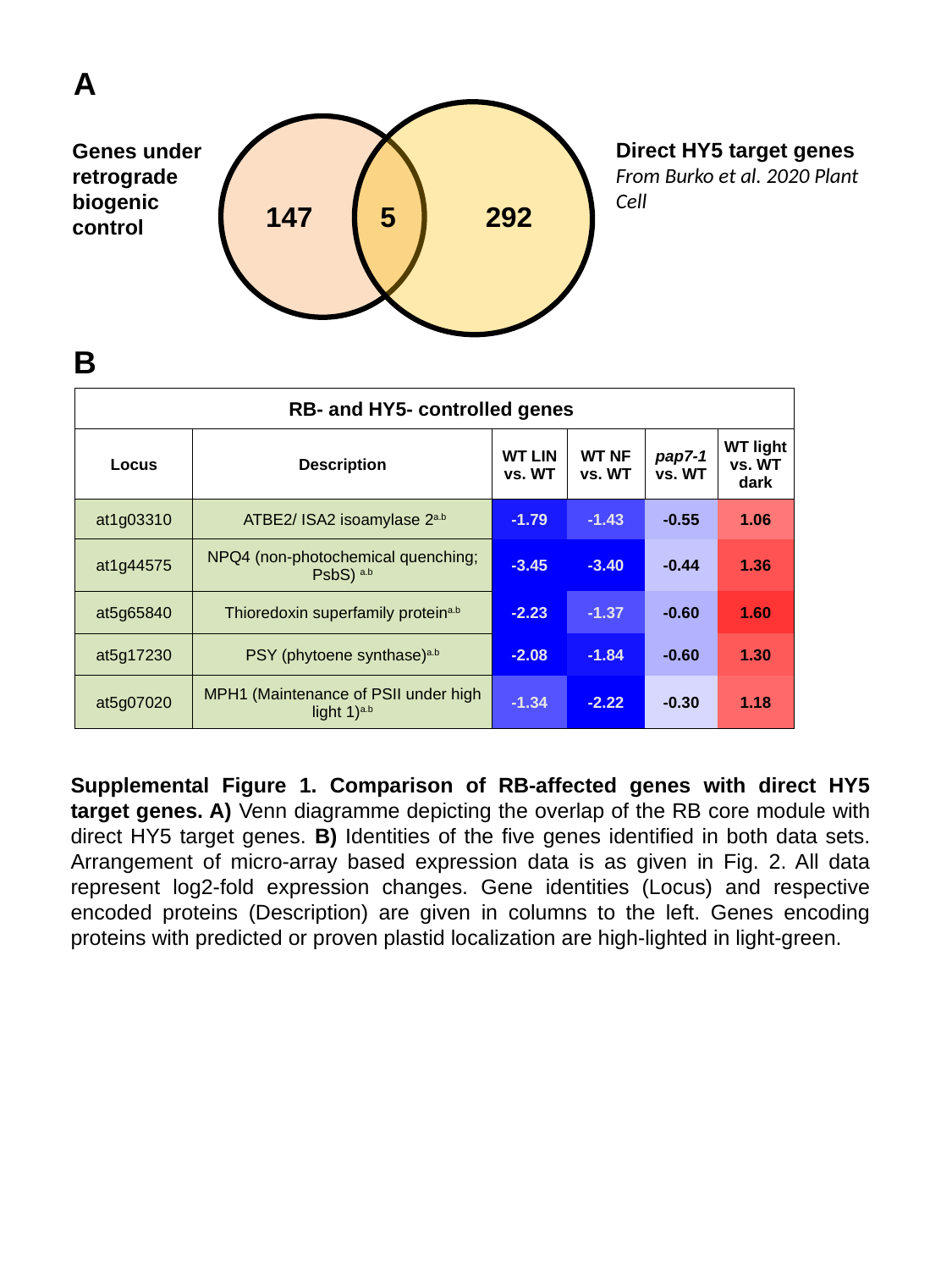

A
Direct HY5 target genes
From Burko et al. 2020 Plant Cell
Genes under retrograde biogenic control
147
5
292
B
| RB- and HY5- controlled genes | | | | | |
| --- | --- | --- | --- | --- | --- |
| Locus | Description | WT LIN vs. WT | WT NF vs. WT | pap7-1 vs. WT | WT light vs. WT dark |
| at1g03310 | ATBE2/ ISA2 isoamylase 2a.b | -1.79 | -1.43 | -0.55 | 1.06 |
| at1g44575 | NPQ4 (non-photochemical quenching; PsbS) a.b | -3.45 | -3.40 | -0.44 | 1.36 |
| at5g65840 | Thioredoxin superfamily proteina.b | -2.23 | -1.37 | -0.60 | 1.60 |
| at5g17230 | PSY (phytoene synthase)a.b | -2.08 | -1.84 | -0.60 | 1.30 |
| at5g07020 | MPH1 (Maintenance of PSII under high light 1)a.b | -1.34 | -2.22 | -0.30 | 1.18 |
Supplemental Figure 1. Comparison of RB-affected genes with direct HY5 target genes. A) Venn diagramme depicting the overlap of the RB core module with direct HY5 target genes. B) Identities of the five genes identified in both data sets. Arrangement of micro-array based expression data is as given in Fig. 2. All data represent log2-fold expression changes. Gene identities (Locus) and respective encoded proteins (Description) are given in columns to the left. Genes encoding proteins with predicted or proven plastid localization are high-lighted in light-green.

## Slide 2
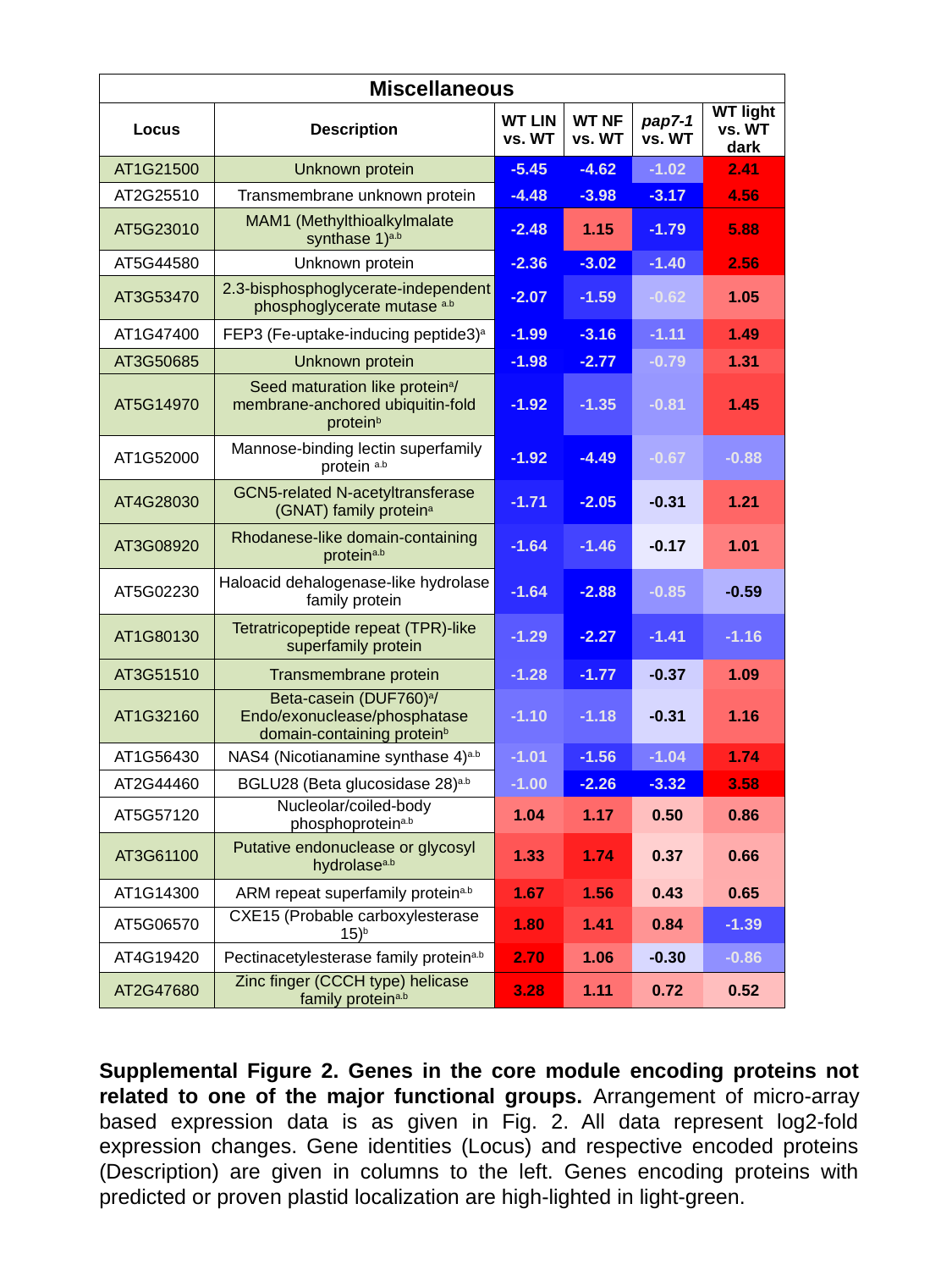

| Miscellaneous | | | | | |
| --- | --- | --- | --- | --- | --- |
| Locus | Description | WT LIN vs. WT | WT NF vs. WT | pap7-1 vs. WT | WT light vs. WT dark |
| AT1G21500 | Unknown protein | -5.45 | -4.62 | -1.02 | 2.41 |
| AT2G25510 | Transmembrane unknown protein | -4.48 | -3.98 | -3.17 | 4.56 |
| AT5G23010 | MAM1 (Methylthioalkylmalate synthase 1)a.b | -2.48 | 1.15 | -1.79 | 5.88 |
| AT5G44580 | Unknown protein | -2.36 | -3.02 | -1.40 | 2.56 |
| AT3G53470 | 2.3-bisphosphoglycerate-independent phosphoglycerate mutase a.b | -2.07 | -1.59 | -0.62 | 1.05 |
| AT1G47400 | FEP3 (Fe-uptake-inducing peptide3)a | -1.99 | -3.16 | -1.11 | 1.49 |
| AT3G50685 | Unknown protein | -1.98 | -2.77 | -0.79 | 1.31 |
| AT5G14970 | Seed maturation like proteina/ membrane-anchored ubiquitin-fold proteinb | -1.92 | -1.35 | -0.81 | 1.45 |
| AT1G52000 | Mannose-binding lectin superfamily protein a.b | -1.92 | -4.49 | -0.67 | -0.88 |
| AT4G28030 | GCN5-related N-acetyltransferase (GNAT) family proteina | -1.71 | -2.05 | -0.31 | 1.21 |
| AT3G08920 | Rhodanese-like domain-containing proteina.b | -1.64 | -1.46 | -0.17 | 1.01 |
| AT5G02230 | Haloacid dehalogenase-like hydrolase family protein | -1.64 | -2.88 | -0.85 | -0.59 |
| AT1G80130 | Tetratricopeptide repeat (TPR)-like superfamily protein | -1.29 | -2.27 | -1.41 | -1.16 |
| AT3G51510 | Transmembrane protein | -1.28 | -1.77 | -0.37 | 1.09 |
| AT1G32160 | Beta-casein (DUF760)a/ Endo/exonuclease/phosphatase domain-containing proteinb | -1.10 | -1.18 | -0.31 | 1.16 |
| AT1G56430 | NAS4 (Nicotianamine synthase 4)a.b | -1.01 | -1.56 | -1.04 | 1.74 |
| AT2G44460 | BGLU28 (Beta glucosidase 28)a.b | -1.00 | -2.26 | -3.32 | 3.58 |
| AT5G57120 | Nucleolar/coiled-body phosphoproteina.b | 1.04 | 1.17 | 0.50 | 0.86 |
| AT3G61100 | Putative endonuclease or glycosyl hydrolasea.b | 1.33 | 1.74 | 0.37 | 0.66 |
| AT1G14300 | ARM repeat superfamily proteina.b | 1.67 | 1.56 | 0.43 | 0.65 |
| AT5G06570 | CXE15 (Probable carboxylesterase 15)b | 1.80 | 1.41 | 0.84 | -1.39 |
| AT4G19420 | Pectinacetylesterase family proteina.b | 2.70 | 1.06 | -0.30 | -0.86 |
| AT2G47680 | Zinc finger (CCCH type) helicase family proteina.b | 3.28 | 1.11 | 0.72 | 0.52 |
Supplemental Figure 2. Genes in the core module encoding proteins not related to one of the major functional groups. Arrangement of micro-array based expression data is as given in Fig. 2. All data represent log2-fold expression changes. Gene identities (Locus) and respective encoded proteins (Description) are given in columns to the left. Genes encoding proteins with predicted or proven plastid localization are high-lighted in light-green.

## Slide 3
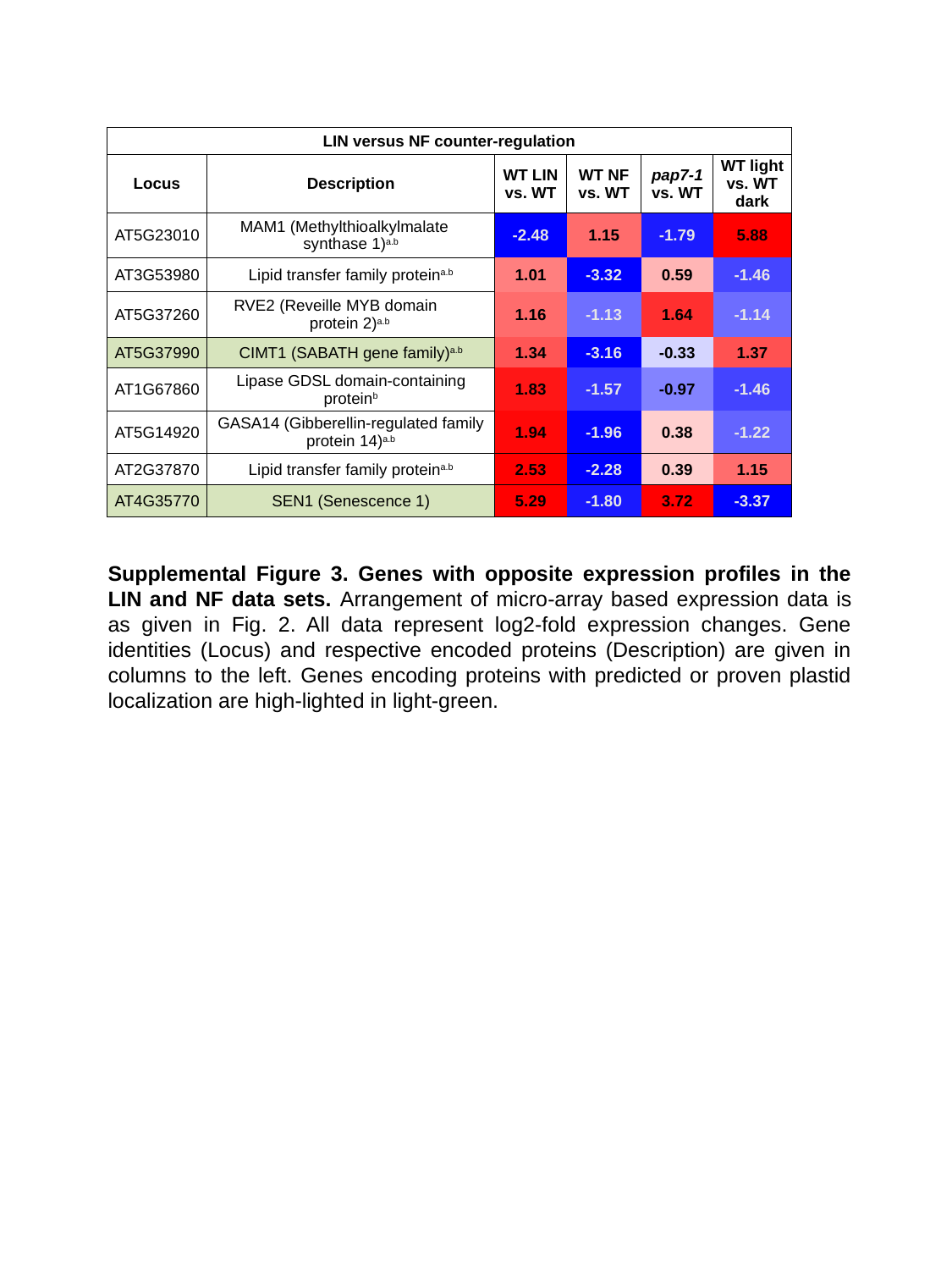

| LIN versus NF counter-regulation | | | | | |
| --- | --- | --- | --- | --- | --- |
| Locus | Description | WT LIN vs. WT | WT NF vs. WT | pap7-1 vs. WT | WT light vs. WT dark |
| AT5G23010 | MAM1 (Methylthioalkylmalate synthase 1)a.b | -2.48 | 1.15 | -1.79 | 5.88 |
| AT3G53980 | Lipid transfer family proteina.b | 1.01 | -3.32 | 0.59 | -1.46 |
| AT5G37260 | RVE2 (Reveille MYB domain protein 2)a.b | 1.16 | -1.13 | 1.64 | -1.14 |
| AT5G37990 | CIMT1 (SABATH gene family)a.b | 1.34 | -3.16 | -0.33 | 1.37 |
| AT1G67860 | Lipase GDSL domain-containing proteinb | 1.83 | -1.57 | -0.97 | -1.46 |
| AT5G14920 | GASA14 (Gibberellin-regulated family protein 14)a.b | 1.94 | -1.96 | 0.38 | -1.22 |
| AT2G37870 | Lipid transfer family proteina.b | 2.53 | -2.28 | 0.39 | 1.15 |
| AT4G35770 | SEN1 (Senescence 1) | 5.29 | -1.80 | 3.72 | -3.37 |
Supplemental Figure 3. Genes with opposite expression profiles in the LIN and NF data sets. Arrangement of micro-array based expression data is as given in Fig. 2. All data represent log2-fold expression changes. Gene identities (Locus) and respective encoded proteins (Description) are given in columns to the left. Genes encoding proteins with predicted or proven plastid localization are high-lighted in light-green.
